# Cardiac Myocyte Diversity and a Fibroblast Network in the Junctional Region of the Zebrafish Heart Revealed by Transmission and Serial Block-Face Scanning Electron Microscopy

**DOI:** 10.1371/journal.pone.0072388

**Published:** 2013-08-23

**Authors:** Pascal J. Lafontant, Ali R. Behzad, Evelyn Brown, Paul Landry, Norman Hu, Alan R. Burns

**Affiliations:** 1 Department of Biology, DePauw University, Greencastle, Indiana, United States of America; 2 Imaging and Characterization Core Lab, King Abdullah University of Science and Technology, Thuwal, Kingdom of Saudi Arabia; 3 College of Optometry, University of Houston, Houston, Texas, United States of America; 4 Department of Pediatrics, University of Utah School of Medicine, Salt Lake City, Utah, United States of America; 5 Department of Pediatrics, Baylor College of Medicine, Houston, Texas, United States of America; New York Medical College, United States of America

## Abstract

The zebrafish has emerged as an important model of heart development and regeneration. While the structural characteristics of the developing and adult zebrafish ventricle have been previously studied, little attention has been paid to the nature of the interface between the compact and spongy myocardium. Here we describe how these two distinct layers are structurally and functionally integrated. We demonstrate by transmission electron microscopy that this interface is complex and composed primarily of a junctional region occupied by collagen, as well as a population of fibroblasts that form a highly complex network. We also describe a continuum of uniquely flattened transitional cardiac myocytes that form a circumferential plate upon which the radially-oriented luminal trabeculae are anchored. In addition, we have uncovered within the transitional ring a subpopulation of markedly electron dense cardiac myocytes. At discrete intervals the transitional cardiac myocytes form contact bridges across the junctional space that are stabilized through localized desmosomes and fascia adherentes junctions with adjacent compact cardiac myocytes. Finally using serial block-face scanning electron microscopy, segmentation and volume reconstruction, we confirm the three-dimensional nature of the junctional region as well as the presence of the sheet-like fibroblast network. These ultrastructural studies demonstrate the previously unrecognized complexity with which the compact and spongy layers are structurally integrated, and provide a new basis for understanding development and regeneration in the zebrafish heart.

## Introduction

The zebrafish heart consists of a spongy ventricular myocardium made of trabecular bundles projecting radially into the ventricular lumen, and of an outer compact heart layer that encases the spongy trabeculae. The proportion of spongy to compact heart varies significantly between fish and appears to strongly correlate to each species particular ecological physiology [1], [2], [3], [4]. The hearts of fish with relatively low activity consist primarily or exclusively of a spongy myocardium with a rudimentary compact myocardium fed by deoxygenated luminal blood flow (type-1 heart). More active fish display a thicker compact myocardium invested with vessels carrying oxygenated blood (type-2 heart). The two myocardial segments have been historically and empirically considered distinct anatomical structures that form a functional unit supporting the physiological demands of fish. Because of its hypothesized importance in fish ventricular function, the characteristics of the spongy-compact interface (SCI) between the two ventricular layers have been an area of considerable interest and controversy.

The zebrafish has emerged as an important model of heart development and regeneration. The adult zebrafish heart is a type-2 heart with a compact layer perfused by capillaries. An extensive number of studies have provided insights into the molecular mechanisms underlying the development of the zebrafish ventricle [5], [6], [7] and its spongy trabeculae [8], [9]. Other studies have explored the molecular mechanisms that orchestrate the zebrafish’s remarkable ability to regenerate [10], [11]. More recently, the emergence of the mature zebrafish ventricle has been demonstrated by genetic approach to be driven by clonally-dependent cardiac myocytes, illustrating diversity within the ventricular myocyte population [12]. A number of studies have also provided insight into the ultrastructural characteristics of the developing and adult zebrafish heart [13], [14], [15]. However to date the nature of SCI in the zebrafish heart has received little attention.

Studies in a variety of fish using different approaches have resulted in markedly diverse understandings and models of this interface region. Early studies in the albacore tuna suggested that the two myocardial layers were attached by a connective tissue layer [16], [17]. Transmission electron microscopy studies in four different fish species, not including the tuna, provided the first detailed evidence not only for connective tissue at the interface of the two myocardial layers [18], [19], but of the presence of fibroblasts and a distinct set of flattened transitional cardiac myocytes occupying this complex junctional region (JR). On the other hand, in a more recent study of the sockeye salmon and rainbow trout, similar structures in the interface region were not observed [20]. Whether these studies underscore the structural diversity of the JR in fish, or reflect differences in study methodologies is not clear. The different findings in the salmon and trout heart may be indicative of the intra- and inter-species diversity in the architectural arrangements of the two layers.

The purpose of our study was to ascertain the cellular and ultrastructural nature of the interface region between compact and spongy heart of adult zebrafish using light and transmission electron microcopy (TEM). Here, we provide evidence of a complex and previously unrecognized JR containing a network of fibroblasts, a subpopulation of phenotypically transitional cardiac myocytes at the base of the spongy myocardium, and describe the spatial distribution of adherens junctions linking the spongy and compact layers. The three-dimensional structure of the JR was confirmed by serial block-face scanning electron microscopy (SBF-SEM) and computerized segmentation. To our knowledge, this is the first detailed description of the JR of the zebrafish ventricle, as well as the first ultrastructural 3D reconstruction of this region using SBF-SEM.

## Materials and Methods

### Animals

Zebrafish were obtained from Aquatic Research Organisms (Hampton, NH), and maintained in 10-gallon tanks, with 15 to 20 fish per tank, at 28 degrees Celsius on a 14/10 hour day/night cycle. Experimental procedures were approved by the Committee for the Care and Use of Laboratory Animals at DePauw University.

### Transmission Electron Microscopy

For studies of the myocardium in plastic sections and by transmission electron microcopy tissue samples were processed as described previously [21]. Zebrafish were euthanized using 0.2% MS Tricaine. Once all body and opercula movements had ceased, the heart was removed by grasping and pulling the aorta proximal to the bulbus arteriosus. The hearts were fixed in 100 mM cacodylate buffer containing 2.5% glutaraldehyde overnight at 4 degrees C. The hearts were washed the next day in cacodylate buffer and stored at 4 degrees C for later processing. Hearts were post-fixed in 1% tannic acid and transferred to 1% osmium tetroxide, dehydrated in an acetone series, and then embedded in Embed-812 resin (Electron Microscopy Sciences, Hatsfield, PA). Two-micron thick sagittal sections were cut and toluidine-blue stained for light microscopy analysis. Stained sections were visualized and imaged using a Nikon Optiphot (Nikon Instruments Inc., Melville, NY). For electron microscopy analysis, ultra-thin sections 100 nm thick were cut, set on single slot or 200-mesh copper or nickel grids, and imaged on a Tecnai G2 Spirit BioTWIN electron microscope (FEI Company, Hillsboro, OR). Additional fixed and embedded hearts (n = 9) were obtained from the University of Utah and processed as previously described [14]. Briefly, these hearts were perfusion-fixed with 2.5% glutaraldehyde-2% paraformaldehyde in 0.1 M cacodylate buffer, and 4×10^−4^ g/kg dose of diltiazem for 4 hours. The hearts were then post-fixed in 2% osmium tetroxide, dehydrated, and embedded in Spurr’s epoxy resin. Ultra-thin sections 100 nm thick were cut and mounted on single slot or 200-mesh copper or nickel grids and imaged on a Tecnai G2 Spirit BioTWIN electron microscope (FEI Company, Hillsboro, OR).

### Immunofluorescence Imaging

Following isolation, zebrafish hearts were cryoprotected in 30% sucrose in PBS overnight. The following day, the hearts were embedded in freezing medium (Tissue-tek OCT, Torrance, CA), kept at −80 degrees C and later sectioned using a Leica CM 1900 cryostat (Leica Microsystems, Bannockburn, IL). Ten-micron sagittal sections were fixed for 3 minutes in 4% paraformaldehyde, permeabilized with 0.5% triton X in PBS, blocked with 3% BSA for 2 hours, and immunoreacted overnight with anti-MYH1 antibody or MEF antibody (Santa Cruz Biotechnology Inc., Santa Cruz, CA), followed by Texas-Red conjugated anti-mouse or anti-rabbit secondary antibody. Next, the sections were incubated with FITC-labeled wheat germ agglutinin (WGA, Triticum vulgaris, Sigma-Aldrich, Saint Louis, MO) for 2 hours. Other sections were stained with TRITC-labeled WGA. Following Hoechst staining, sections were imaged on a Zeiss Axio Imager.M (Carl Zeiss Microscopy, Thornwood, NY) or a Leica TCS SP5 confocal microscope (Leica Microsystems, Bannockburn, IL).

### Serial Block-face Scanning Electron Microscopy Imaging

Tissue fixation and embedding were performed using the previously published protocols with some modifications [22]. Briefly, excised zebrafish hearts were fixed in 2.5% glutaraldehyde in 100 mM sodium cacodylate buffer at 4 degrees C overnight. The hearts were rinsed the next day in 100 mM sodium cacodylate. The hearts were post-fixed and heavy metal-contrasted with potassium ferrocyanide, osmium tetroxide, thiocarbohydrazide, uranyl acetate and lead aspartate. Next, the hearts were dehydrated in ethanol followed by acetone and embedded in Durcupan resin. Approximately 2×2 mm, 100 nm thick sections were obtained, mounted on slot grids and surveyed by TEM. Blocks with optimal tissue orientation and easily identifiable landmarks in the region of interest were selected for further trimming and imaging. Serial block-face sectioning (at 50 or 100 nm imaging intervals) and imaging were performed using a Gatan 3View system (Gatan, Pleasanton, CA) mounted in an FEI Quanta FEG 200 SEM (FEI Company, Hillsboro, OR). The SEM was operated at an accelerating voltage of 2.0 kV and 30 pascals pressure in low vacuum mode. A solid state backscatter detector was used to acquire serial section images (stacks of 800, 632, and 500 sections) from three regions of interest. Three-dimensional segmentation and reconstruction of fibroblasts and cardiac myocytes was performed using Amira 5.2 software.

## Results

### I. Electron Translucent and Electron Dense Cardiac Myocytes are Organized into a Transitional Cell Ring

**Figure 1:**
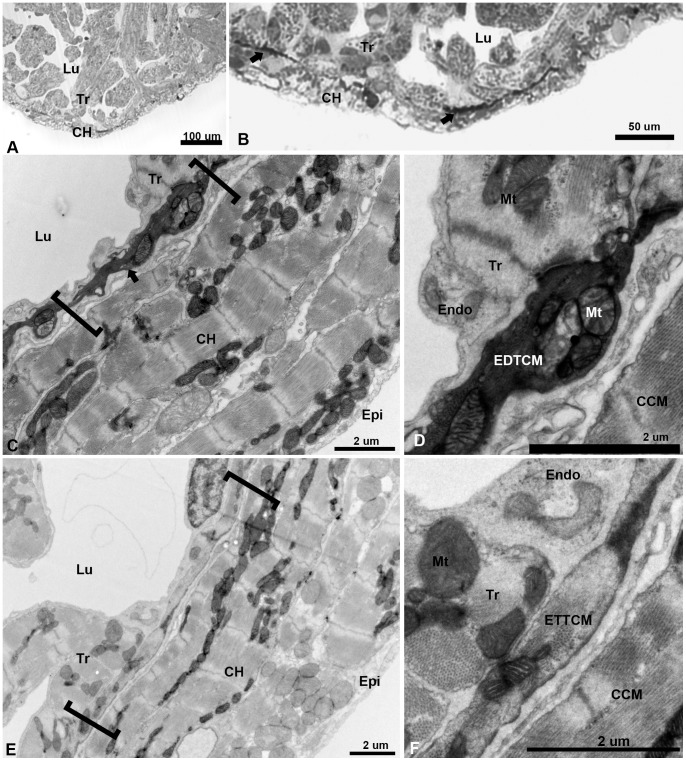
Low magnification of the adult zebrafish heart by light and TEM. (A), light microscopy view of the apical region of the zebrafish ventricle showing a thin layer compact heart (CH) with trabeculae (Tr) projecting within the lumen (Lu). (B), higher magnification of the apex showing darkly stained linear structures (arrows) close to the spongy-compact interface of the ventricular myocardium. (C), low magnification TEM of compact ventricular myocardium constituted of four to five overlapping cardiac myocytes (CM) layers from epicardium (Epi) to lumen (Lu), with electron dense cells (arrow) within a complex junctional region (JR, brackets) at the interface of compact and spongy myocardium. (D), higher magnification show mitochondria (Mt) filled section of electron dense transitional cardiac myocyte (EDTCM). EDTCM are apposed to trabecular cardiac myocytes and endocardial cells (Endo) on the luminal side. EDTCMs are separated from the compact cardiac myocytes (CCM) by a junctional space, an interstitial space within the JR. (E), low magnification TEM of the JR (brackets) showing flattened electron translucent transitional cardiac myocytes (ETTCM) in contact with trabeculae and in proximity to endocardium (Endo) on the luminal side. (F), higher magnification of a flattened cells show presence of actin and myosin filaments, apposition to trabeculae and endocardial cells as well as separation from the compact myocardium by the junctional space.

To determine the nature of the interface region between the compact and spongy heart of the adult zebrafish, we first studied the ventricle under light microscopy. Using two-micron thick toluidine blue-stained plastic embedded sections, we observed the expected organization: a relatively thin layer of the compact heart (approximately 10–20 um thick) framing the trabeculae which projected radially into the ventricular lumen (Fig. 1. A). However closer inspection revealed an unexpected number of darkly-stained elongated structures, at irregular intervals at the interface between the compact and spongy segments of the ventricle (Fig. 1. B). To elucidate the nature of these darkly-stained structures, we studied their characteristics and precise location within the zebrafish junctional region (JR) using transmission electron microscopy (TEM). Low magnification TEM reveals a compact myocardium of 4–5 layers of cardiac myocytes (CM) (Fig. 1. C). The compact and spongy interface is revealed as a complex JR containing a set of flattened CM as compared to the compact or trabecular CM. Ultrastructurally, the darkly stained structures appear as electron dense CM running along the adjacent base of the trabeculae (Fig. 1. D). We have called these cells: “dark cells” or electron dense transitional cardiac myocytes (EDTCM). Except for their high electron density, these cells display similar flattened and rectangular phenotypes as the transitional CM described previously [18]. In adjacent regions, the ventricle displays a similar arrangement but with electron translucent transitional cardiac myocytes (ETTCM) (Fig. 1. E). Actin-myosin filament bundles were easily identified within the ETTCM confirming their CM phenotype (Fig. 1 F), whereas the cytoskeletal contents of the EDTCM were obscured by the electron dense material contained in these cells.

### II. Transitional Cell Ring and Trabeculae Contacts

**Figure 2:**
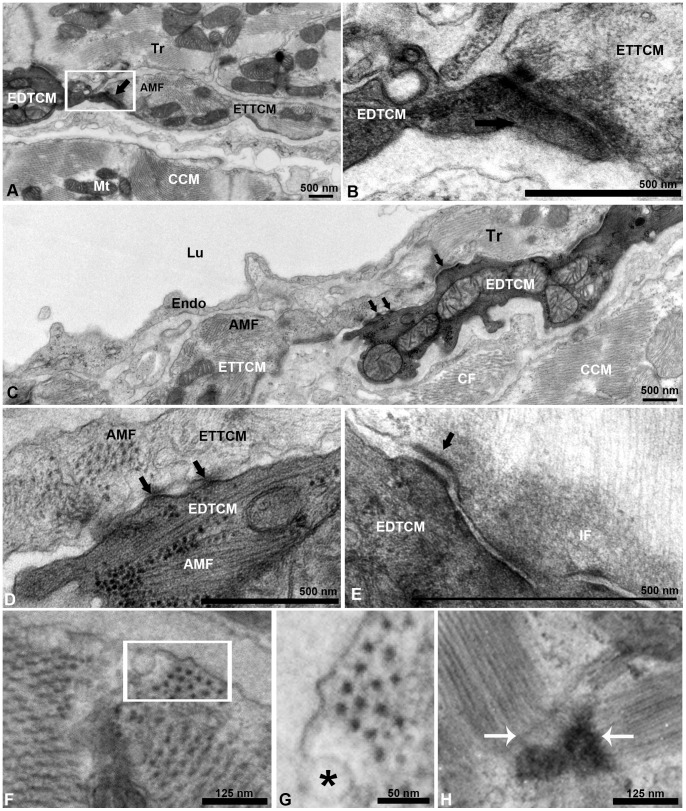
Electron dense and electron translucent transitional cardiac myocytes interactions. (A), example of end-to-end interaction between EDTCM and electron translucent cardiac myocytes (ETTCM) forming a continuum at the spongy-compact interface. (B), higher magnification of contact area with complex adhesion junctions including desmosomes between the two cells. (C), example of interaction between mitochondria filled and actin-myosin filament (AMF) containing EDTCM cells and ETTCM via adhesion junctions (arrows). (D), higher magnification of EDTCM and ETTCM showing gap junctions (arrows), and (E), desmosomes. (F), AMF in a transitional cell. (G), higher magnification (inset in F, rotated) showing the hexagonal array of myosin in cardiac myocytes. (F) sarcomere and z-band in the same cell. AMF, actin-myosin filament; CCM, compact cardiac myocytes, CM, cardiac myocytes; EDTCM, electron dense transitional cardiac myocytes; ETTCM, electron translucent transitional cardiac myocytes; Endo, endocardium; Epi; IF, intermediate filaments; Lu, lumen; Mt, mitochondrion; Tr, trabeculae.

**Figure 3:**
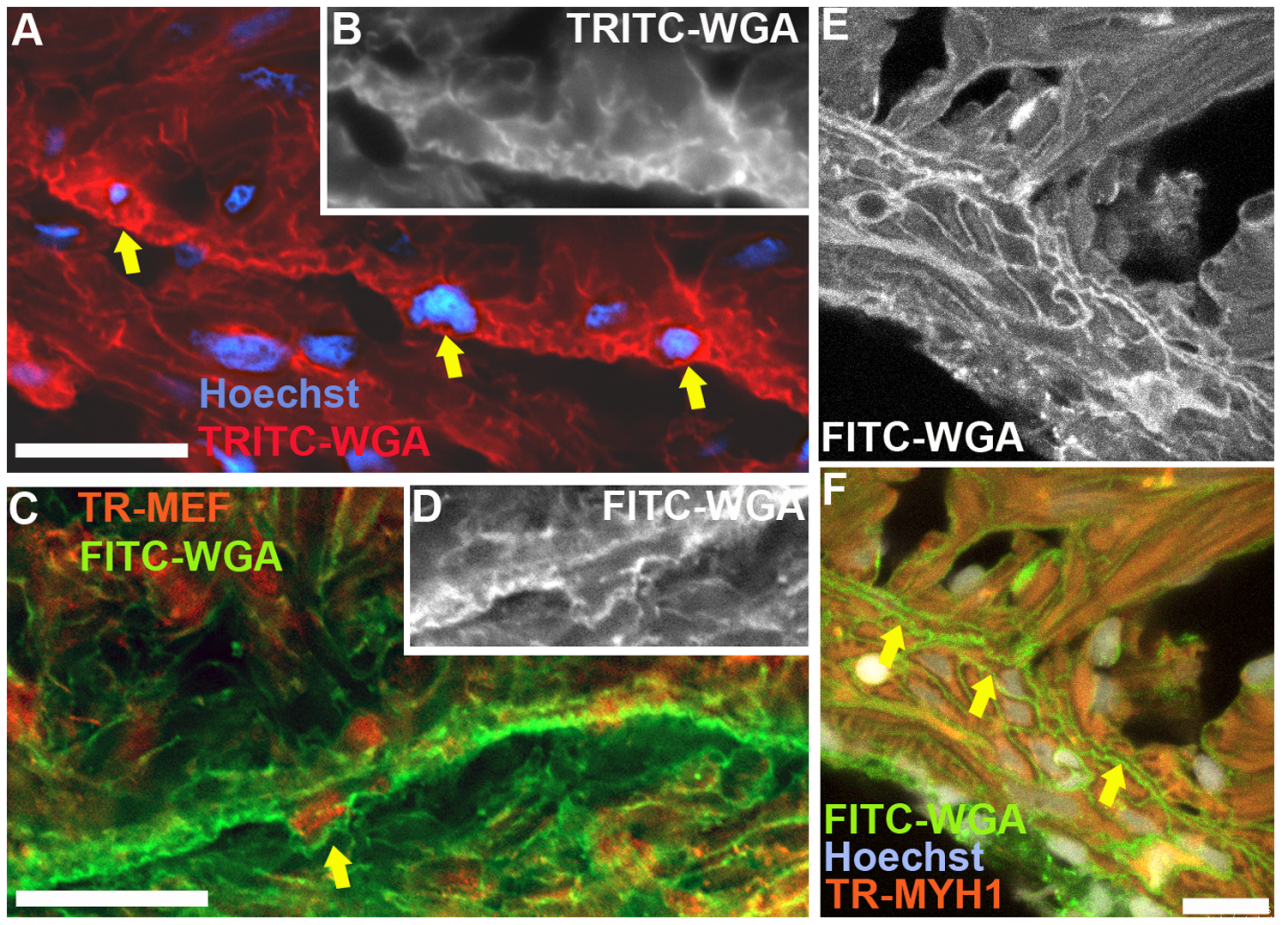
Immunofluorescence imaging of the zebrafish ventricle junctional region. (A), staining with TRITC-labeled wheat germ agglutinin (WGA) highlighting cardiac myocytes borders, in conjunction with Hoechst staining, to illustrate the transitional cardiac myocytes (arrows). (B), original monochrome image of the TRITC-WGA stained cell borders containing the two right-most nuclei identified by the arrows in panel A. (C), immunostaining with MEF antibody to label myocyte nuclei and FITC-labeled WGA-stained cell membranes; the arrow identifies a transitional cardiac myocyte. (D), original monochrome image of the FITC-WGA stained cell membranes in panel C around the nucleus identified by the arrow. (E), confocal image of FITC-WGA-stained cardiac myocyte membranes. (F), overlay images of FITC-WGA in panel E with MYH1 (anti-myosin heavy chain-1 antibody) immunostaining, and Hoechst staining identifying transitional cardiac myocytes (arrows). Scale bar, 10 um.

The EDTCM were in direct contact with the ETTCM (Fig. 2. A). Within the plane of observation, shorter segments of EDTCM alternated with longer segments of ETTCMs, and together these two cell populations appear to form a continuous ring of transitional CM within the JR. The transitional CM could be seen linked at their tapered ends through complex adherent junctions resembling fascia adherentes (Fig. 2. B). Additionally, the transitional CM were in direct contact or in close apposition to the base of CM sheets of projecting spongy trabeculae (Fig. 2. A, C), and in the intertrabecular lacunar spaces they were bounded in their luminal aspect by the endocardium (Fig. 2. C). However in contrast to their connections to trabeculae, no contacts were observed between transitional CM of the transitional ring and the adjacent endocardial cells. In some instances, EDTCM and ETTCM minimally overlapped at their tapered ends (Fig. 2 C), with connections occurring through discrete gap junctions (Fig. 2. D) and desmosomes (Fig. 2. E). Detailed observations of the cytoplasm of these cells show the presence of actin-myosin filaments oriented in different planes (Fig. 2. F). The presence of caveolae and the hexagonal array of equidistant thick myosin filaments characteristic of cardiac muscle cells can be appreciated (Fig. 2. G). Z-bands bordering sarcomeres oriented perpendicularly or obliquely in the plane of observation (Fig. 2. H) can also be seen in these cells, further confirming their cardiac myocyte phenotype. Finally the cardiac nature of these narrow width transitional cells was also confirmed using immunostaining with anti-myosin heavy chain-1 (MYH1) and anti-myocyte enhancer factor-2 (MEF) antibodies to specifically identify cardiac myocytes (Fig. 3).

**Figure 4:**
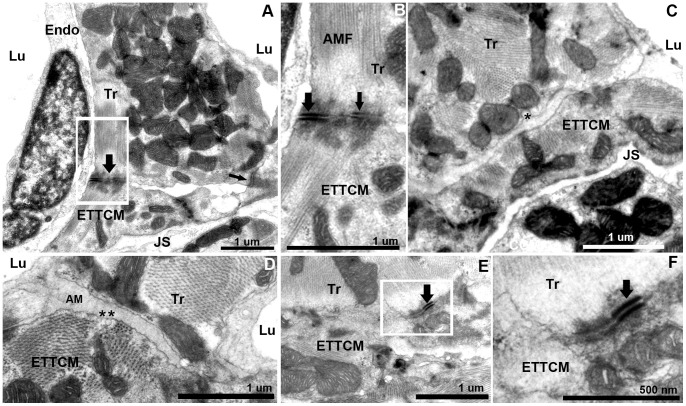
Transitional cardiac myocytes ring and trabecular cardiac myocytes contacts. (A), trabecular cardiac myocytes (Tr) in direct contact with two CM of the transitional ring. Trabeculae and transitional CMs are quasi-perpendicularly oriented. Contacts are mediated by electron dense adhesion junctions (arrows). (B), higher magnification of (A), with fascia adherentes junction (thick arrow) and desmosome (thin arrow) between the Tr and ETTCM. Well organized actin and myosin filaments are oriented at an approximately 145 degree angle. (C, D), region of approximation of trabecular CM and transitional CM with amorphous material (AM) interposed between the two cell membranes. Note a number of caveolae in the trabecular CM membrane (C,*) on their abluminal side, and in the transitional CM membrane (D,*) on the luminal side. (E), another example of desmosome linking a trabecular to a transitional CM. (F), higher magnification of (E).

We further investigated the nature of the arrangement between the transitional CM ring and the CM forming the trabeculae. Transitional CM can be found linked to trabecular myocytes through a variety of adherens junctions. These junctions included fascia adherentes (Fig. 4. A, B) that link actin-myosin filaments of adjacent cells. Various amounts of amorphous material were observed along transitional CM and trabecular CM areas of apposition (Fig. 4 C, D). However discrete electron dense areas with a sub-domain containing desmosomes were also observed (Fig. 4 E, F).

### III. Fibroblast Network and Collagen in the Junctional Region

**Figure 5:**
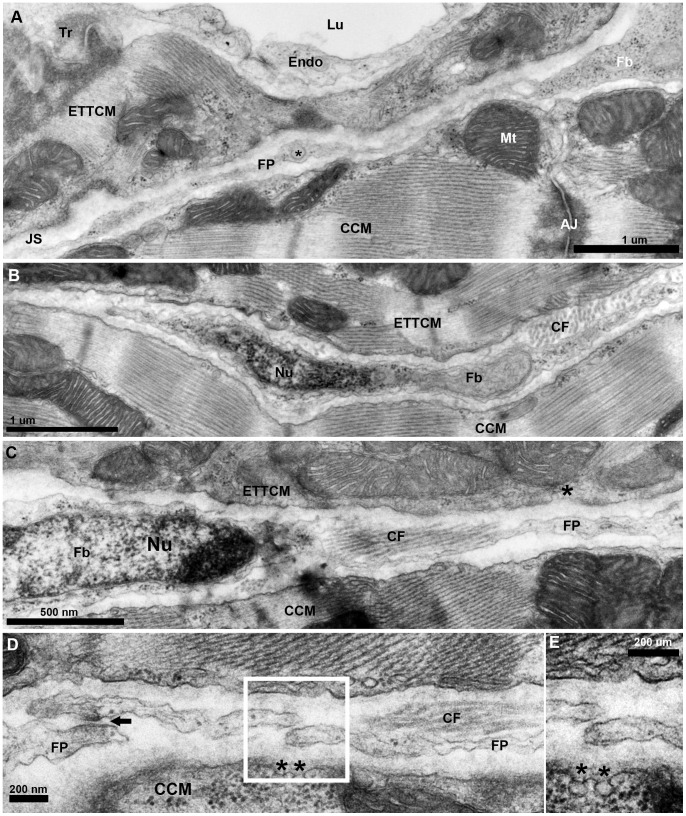
Fibroblasts network in the junctional region of the zebrafish ventricle. (A), TEM of a fibroblast (Fb) cytoplasm and its thin extended process/filopodium (FP) 100 to 200 nm thick spanning the junctional space (JS) in between the transitional CM ring and the adjacent compact cardiac myocyte (CCM). Note vesicle (*) in an enlarged region (∼300 nm) of the fibroblasts filopodium (FP). (B), fibroblast profile with nucleus (Nu) and its cytoplasm extending into a long thin process, and collagen fiber (CF) bundles running perpendicular to the section’s plane. (C), another fibroblast and its nucleus (Nu) and with cytoplasm extending into a long process, and collagen fiber (CF) bundles running parallel to the section’s plane. (D), fibroblast filopodial termini in close approximation. Electron dense region in the close approximation area suggestive of specialized adhesion junction (arrow). Note the presence of caveolae (*) on the membrane of the CCM facing the fibroblast in the junctional space.

The transitional ring of myocytes on its abluminal side is bounded by a narrow (0.5–1 um) connective tissue space. This space separates the EDTCM and ETTCM from the adjacent CM of the compact layer, creating a junctional space within the JR (Fig. 5. A). The junctional space appears to create an extensive structural discontinuity between the spongy and the compact myocardium. It contains numerous cells arranged longitudinally and running parallel to the transitional CM ring. The absence of basement membranes, their elongated shape, and their location within this interstitial space suggest they are fibroblasts (Fig. 5. B, C). These fibroblasts contained ovoid nuclei with scant perinuclear cytoplasm that appeared mostly devoid of organelles, except for occasional vesicles and polyribosomes. In the perinuclear region of the fibroblasts, the junctional space widens to 2–3 microns. The cytoplasm of these fibroblasts is stretched bi-directionally into long thin filopodia-like processes. These fibroblasts mostly form a single layer in the junctional space with little to minimal overlap of their cytoplasm. The fibroblasts and their cytoplasmic processes fit tightly within the junctional space, however a measurable space was always observed between the fibroblast and the adjacent transitional and the compact ventricular CM.

The fibroblasts were also found in close proximity or in direct contact with parallel or perpendicularly-oriented collagen fibers (Fig. 5. B, C). At irregular intervals, terminal profiles displaying long cytoplasmic extensions were in close proximity with the membranes of long cytoplasmic processes originating from another fibroblast (Fig. 5. D). In some instances, cytoplasmic terminal ends of fibroblasts overlapped, or could be seen in contact with the same bundle of collagen. Occasionally fibroblasts terminal ends were found in close contact; however we could not unequivocally confirm specialized adhesion junctions between interacting terminal ends. Occasionally close approximation between fibroblast filipodia and CM could be seen; however no direct contact between fibroblasts and CM and no specialized myocytes-fibroblasts junctions were observed. Close observation of many of the transitional CM however revealed the presence of caveolae within the membranes orientated toward the fibroblasts within the junctional space (Fig. 5. C). Across the junctional space, the compact CM membranes were observed to have numerous caveolae facing toward the fibroblasts (Fig. 5. D, E). More examples of caveolae could be seen on the membranes of CM facing the junctional space at a distance from the fibroblast processes (Figure S1). While most fibroblast nuclei and cytoplasmic processes resided within the JR, some cytoplasmic projections could be seen invested in the compact myocardium adjacent to small vessels, suggesting that fibroblasts may not be locally restricted to the junctional space.

### IV. Transitional Cardiac Myocytes Contacts across the Junctional Space

**Figure 6:**
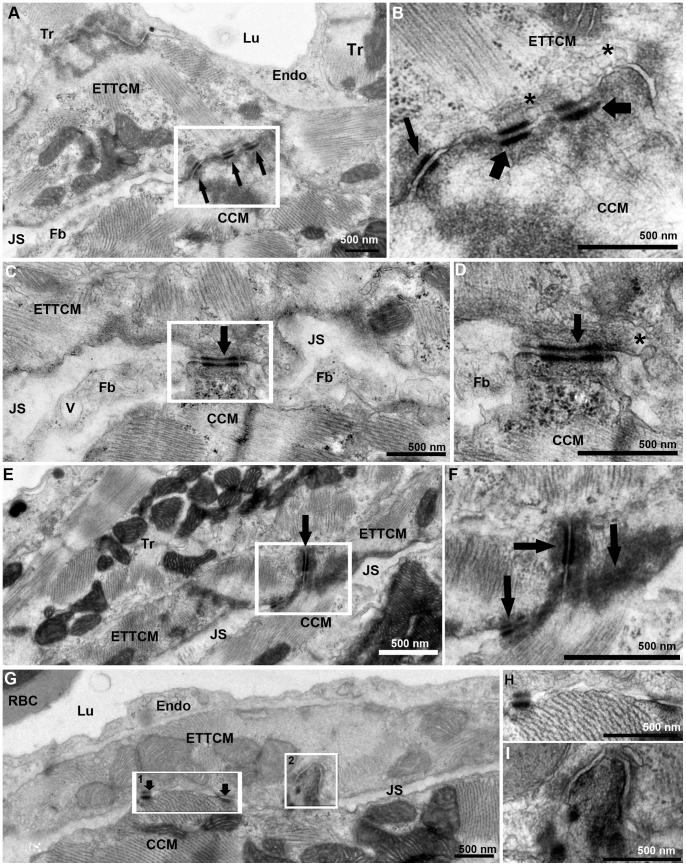
Transitional CM ring and compact CM contacts. (A), ETTCM and Compact CM direct contacts mediated by adhesion junctions (arrows). Note the JS with fibroblast (Fb) on the right and narrow JS of the left of the contact region. (B), higher magnification of inset in (A) showing adhesion junctions associated with fascia adherentes (thick arrow), and also with desmosomes (thin arrow). Note the abundance of caveolae (*) in the transitional CM membrane in the contact region. (C), another narrow region of contact (arrow) between a transitional and a compact cardiac myocyte forming a bridge between the two cells and adjacent Z-band in the CCM. A fibroblast process is seen on each side of the junction. Note a vesicle (V) in the fibroblast process. (D), higher magnification of the contact area (arrow) showing a desmosome associated with actin, myosin, and intermediate filaments on the ETTCM and abundant ribosomes (rb) in the CCM. (E), a tri-cellular junction between two transitional cardiac CM and one compact CM. (F), higher magnification of (E, inset) showing desmosomes between the ETTCM and each ETTCM with the compact CM (arrows). (G), discrete adhesion junction between a transitional and compact CM (arrows). (H), higher magnification of inset 1 of (F). (I), higher magnification of inset 2 of F, showing compact CM contact within an invagination of the transitional CM.

From our observations, we estimated that the junctional space containing fibroblasts and collagen occupies 80 to 90% of the junctional region between compact and spongy heart, so that the majority of the CM membranes in the transitional ring are not directly linked to the compact heart. The remaining portion of the spongy-compact interface consisted of cell membrane appositions between transitional CM and the most adjacent CM of the compact heart (Fig. 6. A). These regions of apposition contained specialized adherens junctions including closely spaced desmosomes and fascia adherents (Fig. 6. B). These areas of contact between the transitional ring and compact myocardium circumscribed the junctional space and interrupted the course of the fibroblasts and their filopodia in the plane of the sections (Fig. 6. C, D). Occasionally a region of tri-cellular contact was observed, where a compact myocyte was joined at the end-to-end contact of two transitional CM through desmosomes (Fig. 6. E, F). However most transitional ring myocytes and compact CM contacts occurred through apposition of their lateral borders via desmosomes (Fig. 6. G, H), membrane protrusions, and interdigitations (Fig. 6. I).

### V. Serial Block-face SEM, Segmentation, and Volume Reconstruction of the Junctional Region

**Figure 7:**
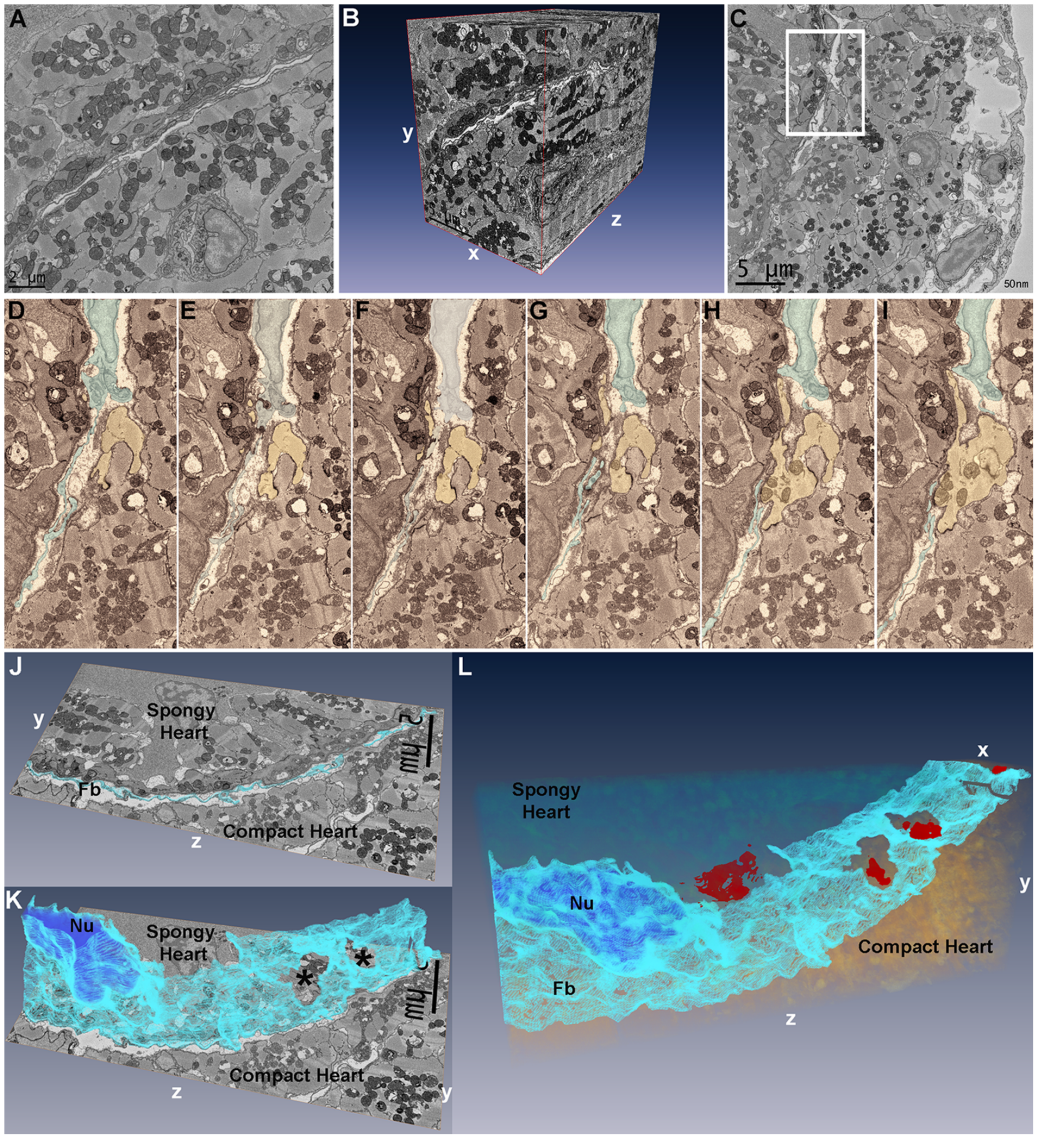
Segmentation and three-dimensional reconstruction of the junctional region of zebrafish ventricle. (A), the first 25×25 um image of 800 images acquired in a region of interest with fibroblasts running diagonally across the image plane, and a vessel profile in the lower half. (B), three-dimensional reconstruction using Amira 5.2 of 800 25×25 um sections at 50 nm intervals demonstrating the 3D spread of fibroblasts in the junctional space. The vessel in Panel A is seen projected in the yz plane in the compact myocardium. (C), a single 25×25 um image of 500 images from another region of interest with inset containing a fibroblast nucleus and fibroblasts processes within the junctional space. (D–I), six serial sections of panel C inset region, 100 nm apart, with fibroblasts (highlighted, blue) in the junctional space and a cardiac myocyte (yellow) from the compact heart. (D–F), fibroblasts can be seen occupying the uninterrupted junctional space. (G), shows the cardiac myocyte progressively protruding into the junctional space while fibroblasts processes are parted in the plane of observation. (H–I), the compact heart cardiac myocyte makes contact with the spongy heart cardiac myocyte. (J), single section in the yz plane with fibroblasts (Fb) highlighted in light blue. (K), following segmentation of 60 serial sections in Amira, 3D reconstruction of fibroblasts (light blue) demonstrates their sheet-like structure; the segmented nucleus (Nu) can be seen (dark blue). Two openings are revealed in the fibroblasts sheet (*); these openings correspond to the location of compact-transitional ring myocytes contacts. (L), Additional segmentation of the transitional cardiac myocytes in 25×25×6 um volume, showing the sheet-like appearance of fibroblasts within the junctional region. Four contact surfaces between compact and transitional cardiac myocytes are revealed (red) through openings in the fibroblasts network.

To better assess the three-dimensional architecture of the junctional region and confirm the three-dimensional nature of the fibroblast network, we performed serial block-face scanning electron microcopy (SBF-SEM) of the adult zebrafish heart. A preparatory survey of the fibroblast network in the JR of the zebrafish ventricle was performed by TEM in 100 nm sections from heavy metal-contrasted Durcupan-embedded hearts. The junctional region and the fibroblasts were easily identifiable. Image stacks generated with the FEI Quanta 200 FEG SEM equipped with the Gatan 3View (Fig. 7. A) were used to generate three-dimensional reconstructions of the JR architecture (Fig. 7. B, Movie S1), and confirm the 3D projection of fibroblasts in the junctional volume space. Observation of a subset of serial sections demonstrated the predicted discontinuous nature of cellular contacts between the compact heart and transitional myocytes that anchor the luminal trabeculae, and confirmed fibroblast–fibroblast contacts in the junctional space (Fig. 7, C–I, Movie S2). Segmentation of fibroblasts from serial sections followed by three-dimensional reconstruction demonstrated the sheet-like nature of fibroblasts arranged in a network occupying the junctional space of the zebrafish heart (Fig. 7. J, K). Additional segmentation of transitional and compact cardiac myocytes demonstrated contact surfaces between compact and spongy heart through the fibroblast network layer (Fig. 7, L).

## Discussion

This paper documents a previously unrecognized connective tissue space located at the interface of the spongy and compact myocardial of the adult zebrafish heart. It extends our understanding of the cellular and ultrastructural nature of the ventricular JR of an important research model. The existence of a connective tissue layer in the junctional region of type-2 fish hearts has remained controversial. A continuous fibrous membrane interposed between the compact and spongy layers has been reported in the hearts of tuna [16], [17], atlantic salmon and rainbow trout [23]. These observations led to the hypothesis of the connective tissue as an important adhesive substrate between the two myocardial layers. In these early studies, detailed composition of the connective tissue layer was not described. Midttun using TEM demonstrated in four type-2 fish hearts species the presence of fibroblasts and collagen fibers within a 6 to 7 um wide space existing in the junctional region [18]. By contrast in a study by Pieperhoff et al., using light and scanning electron microscopy, a connective tissue layer at the interface of the two myocardial layers in the sockeye salmon and trout was not observed [20], beyond small focal regions of extracellular matrix enrichment. Unlike the previous studies, these authors challenged the hypothesis of a connective tissue layer separating the two myocardial layers. In both Midttun’s and our studies, the junctional space and its content were not readily detected by light microscopy; however, they could be observed by transmission electron microscopy, suggesting that ultrastructural thin sections imaging may be necessary to describe the structure of the JR of fish ventricles.

Our ultrastructural observations reveal a junctional space measuring for the most part less than one micrometer wide, populated by a single layer of fibroblasts organized into a network, and framed by CM belonging the compact myocardium distally and the spongy myocardium proximally. Fibroblasts are an important component of the mammalian heart and play an essential role in heart structure and physiology. They are a source of extracellular matrix molecules and growth factors, and they serve as sensors for mechanical signals [24], [25], [26], [27], [28]. While it has been suggested in early studies that collagen accumulation modulates regeneration in the zebrafish ventricle [10], fibroblasts in the fish heart have received little attention. More recently studies of cardiac injury in the zebrafish [29], [30], [31] and giant danio [21] demonstrated accumulation and resorption of collagen during fish ventricular regeneration, suggesting an important role for fibroblasts. Indeed the presence of activated fibroblasts was observed during ventricular remodeling in the zebrafish [31] and giant danio heart [21]. The presence of fibroblasts has previously been noted in the sub-epicardial space of fish species, including the zebrafish [14]. In the present study, the fibroblasts’ arrangement suggests a highly organized three-dimensional network exists at the interface of the compact and spongy ventricle. The function of this fibroblast network is presently unclear.

Fibroblast networks have been documented in a variety of organs and species, with direct fibroblast-fibroblast contacts being observed in mammalian corneas [32], [33]. Fibroblast interactions suggestive of a network have also been reported in the mammalian heart [34]. Fibroblasts have also been implicated in physiological mammalian cardiac muscle growth [27] as well as in injury responses [35], [36], [37], [38]. Our study by TEM and the reconstruction following segmentation of the SBF-SEM micrographs demonstrate that fibroblasts form a three-dimensional network in the JR of the adult zebrafish heart. In addition to fibroblast-fibroblast interactions, direct CM and fibroblast contacts have also been reported in normal and diseased hearts and in culture [39], [40], [41]. In this study however, we did not uncover direct CM-fibroblasts contacts in the uninjured zebrafish heart. When fibroblast processes were seen in close proximity and in apparent apposition to myocytes, the intervening myocyte basement membrane was always present. Yet we cannot rule out that these contacts exist and may occur at a frequency too low to be easily detected. Nevertheless, we found a remarkable number of caveolae in the sarcolemma of CM bordering the junctional space and facing adjacent fibroblasts. This suggests possible mechanisms by which paracrine signaling may take place between adjacent CM and fibroblasts occupying the junctional space.

The present study also establishes that the junctional space occupies the majority of the overall SCI, and creates an extensive volume of non-contact between the two myocardial layers. Given the sparsity of the collagen fibers observed in the junctional space, we question whether in the zebrafish, the connective tissue would provide sufficient support to maintain the structural integrity of the two layers. We speculate that the connective tissue compartment may not be the primary mechanism by which adhesion between the two myocardia is maintained. Indeed, the junctional space is interrupted by contact bridges between the two layers at discrete intervals through membrane apposition of adjacent CM, and that desmosomes and fascia adherentes are frequently found within the contact regions between transitional zebrafish CM and compact CM. These findings are consistent with the demonstration of highly enriched adhesion junction molecules between the two myocardial layers observed in salmon and trout [20].

An important finding is the transitional ring of cardiac myocytes similar to that previously described by Midtunn, that underscores the phenotypic diversity of cardiac myocytes present in the zebrafish heart, suggesting specific structure-function relationship. The notion of cardiac myocyte diversity is apparent in recent work demonstrating the clonal contribution of cardiac myocytes to the emergence of the zebrafish heart [12]. In that study two populations of peripheral cardiac myocytes were described by genetic labeling and spatio-temporal characteristics: a primordial and a cortical set. The localization of the primordial layer suggests it occupies a space similar to the transitional ring population described in the present study. Consequently, it would be interesting to determine whether these two populations spatially interact or are one and the same. Another intriguing finding is the discovery of a subset of “dark” electron dense myocytes within the transitional CM ring that supports the trabeculae. To our knowledge, these electron dense cardiac myocytes have not been reported in fish or mammalian hearts. A set of dark cells was observed in the ventricle of icefish; however, they were believed to be non-contractile in nature [42]. Observations of the zebrafish electron dense cells at high magnification show clear evidence of myofibrils. In addition these cells form a continuum with adjacent electron translucent CM as well as other electron dense CM in the transitional ring and myocytes of adjacent trabeculae via adhesive junctions. These observations raise the intriguing possibility of further phenotypic specialization within the transitional CM population bordering the JS. We speculate this particular phenotypic change may be regulated by regional stress distribution within the myocardium or may reflect their metabolic state. The understanding of the functional and the molecular differences between the dark and light cells within the transitional ring warrants further study.

### Conclusions

**Figure 8:**
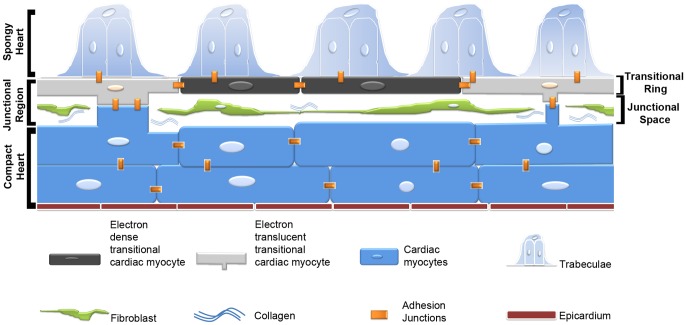
Schematic representation of the interface region between compact and spongy myocardium in the adult zebrafish. The interface consists of a complex junctional region with an interstitial space populated by a fibroblast network and collagen fibrils. A ring of flattened cardiac myocytes forms the base of the projecting luminal trabeculae, and at discrete intervals make contacts with the compact myocardium though bridges across the junctional space. These areas of contact display complex adhesions junctions including desmosomes and fascia adherents that integrate the two myocardia into a structural unit and may mediate electrical and functional integration of the two myocardia. CCM, compact cardiac myocytes; ETTCM, electron translucent transitional cardiac myocytes; EDTCM, electron dense transitional cardiac myocytes.

In conclusion, this study sheds light on the phenotypic diversity of CM at the JR of the adult zebrafish heart. It documents the existence of a fibroblast network that contributes to the architectural complexity of the region (Fig. 8). We suggest the transitional CM form the main anchoring substrate for the trabecular and compact heart. It is possible that in type-2 fish hearts, both the connective tissue layer and the adherens junctions account for the integrity of the spongy-compact interface. It is also possible that in fish, the ratio of connective tissue and direct CM adhesive contact, as well as the distribution of adherens junctions might be dependent on the species, their age and ecological physiology. In the end it is intriguing to consider how the articulation of the compact and spongy interface progresses during development and how the re-articulation occurs during regeneration of the zebrafish heart.

## Supporting Information

Figure S1 **Transitional myocyte abluminal caveolae.** (A), Junctional space (JS) with a fibroblast process (FP). (B), Higher magnification of inset in (A) showing caveolae (*) on transitional CM facing the JS.
(TIF)

Movie S1 **Architecture of the junctional region of zebrafish heart.** The sheet-like nature of fibroblasts can be appreciated.
(MP4)

Movie S2 **Segmented fibroblasts and transitional CM.**
(MP4)
