# Supplementary figures and images for: Cardiac Myocyte Diversity and a Fibroblast Network in the Junctional Region of the Zebrafish Heart Revealed by Transmission and Serial Block-Face Scanning Electron Microscopy
Source: PLoS One. 2013 Aug 23;8(8):e72388. doi: 10.1371/journal.pone.0072388 (PMC3751930; doi:10.1371/journal.pone.0072388)

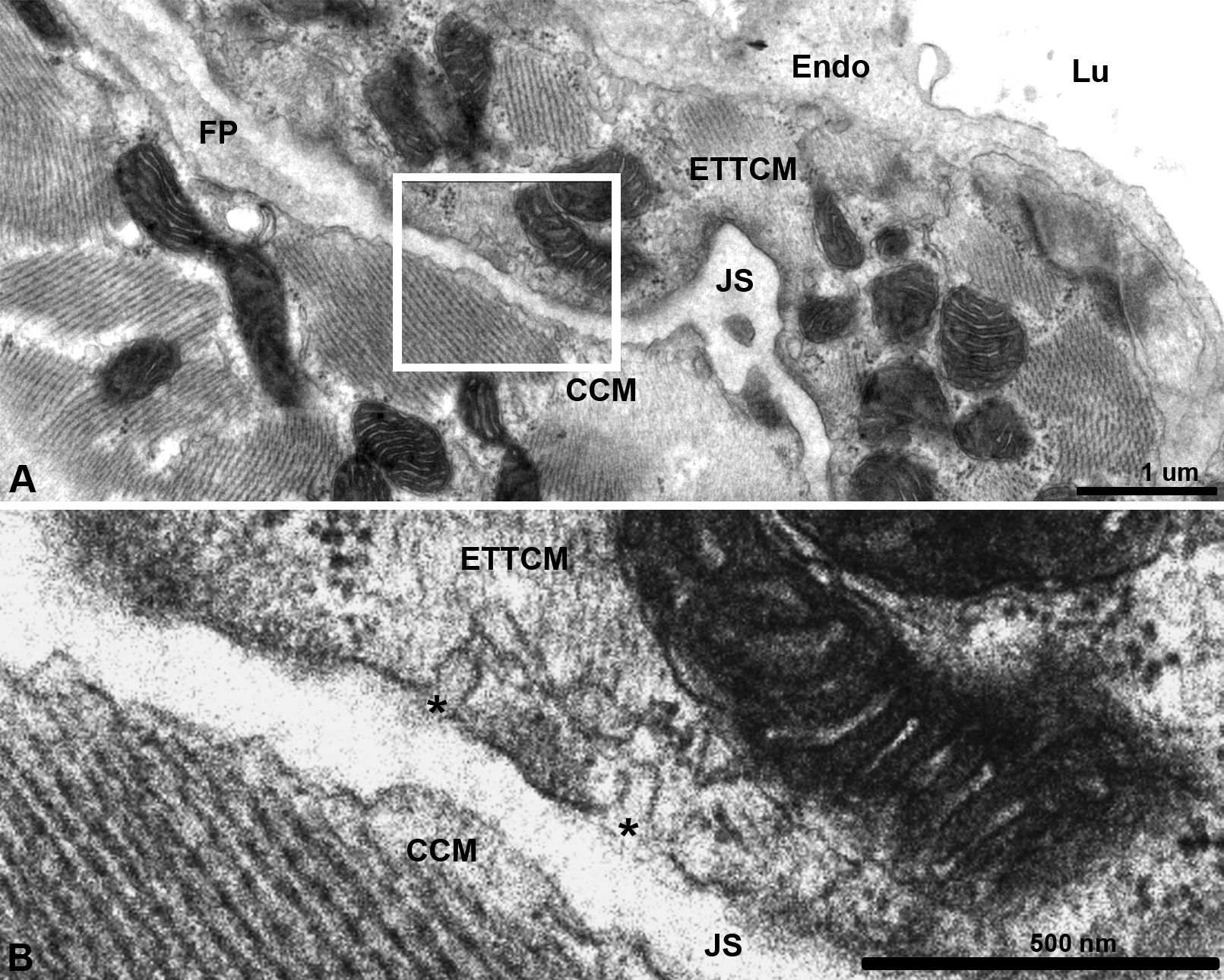

Supplement: Figure S1 — Transitional myocyte abluminal caveolae. (A), Junctional space (JS) with a fibroblast process (FP). (B), Higher magnification of inset in (A) showing caveolae (*) on transitional CM facing the JS. (TIF) [file pone.0072388.s001.tif]
